# Bottom-up approach to strengthen community-based malaria control strategy from community health workers’ perceptions of their past, present, and future: a qualitative study in Palawan, Philippines

**DOI:** 10.1186/s41182-018-0105-x

**Published:** 2018-07-04

**Authors:** Emilie Louise Akiko Matsumoto-Takahashi, Pilarita Tongol-Rivera, Elena Andino Villacorte, Ray Uyaan Angluben, Masamine Jimba, Shigeyuki Kano

**Affiliations:** 1 Department of Tropical Medicine and Malaria, Research Institute, National Center for Global Health and Medicine, 1-21-1 Toyama, Shinjuku-ku, Tokyo, 162-8655 Japan; 2 Department of Community and Global Health, Graduate School of Medicine, The University of Tokyo, 7-3-1 Hongo, Bunkyo-ku, Tokyo, 113-8654 Japan; 3 Department of Parasitology, College of Public Health, University of the Philippines Manila, 625 Pedro Gil Street, Ermita, Manila, Philippines; 4 Pilipinas Shell Foundation, Inc., 5300 Puerto Princesa City, Palawan Philippines

**Keywords:** Malaria, Microscopist, Community health workers, Program development, Program evaluation

## Abstract

**Background:**

Microscopists have active roles in bringing malaria diagnosis and treatment closer to households in Palawan, the highest malaria-endemic province in the Philippines. To accelerate the elimination of malaria in Palawan, we performed a study based on the bottom-up approach to provide profound data to strengthen this community-based malaria control from the microscopists’ point of view.

**Methods:**

We performed a qualitative cross-sectional study in Palawan. Four focus group discussions with 50 microscopists were conducted in Palawan from November 2010 to February 2011. During the discussions, the following open-ended questions were addressed: motivation for applying to be microscopists in the “Past” category; job satisfaction, role, problems, and saddest and happiest experiences working as microscopists in the “Present” category; and willingness towards task shifting in the “Future” category. Data were transcribed and analyzed by framework analysis using the NVivo software program.

**Results:**

The present study innovatively proposed the following strategies: reinforcement strategy (adequate supplies and settings), highly prioritized additional strategies (improving social status of microscopists, issuing a travel budget, and including indigenous populations), regional additional strategies (additional malaria control in the southern region and task shifting in the northern region), and less prioritized additional strategies (employment policy and health checkup).

**Conclusion:**

A bottom-up approach using microscopists’ perceptions would be a valuable method to propose practical and effective additional strategies for strengthening community-based malaria control.

## Background

Palawan has the highest incidence/prevalence of malaria among the endemic provinces of the Philippines, where microscopists, as community health workers (CHWs), have active roles in bringing malaria diagnosis and treatment closer to households to support the limited health care services [1, 2]. In many malaria-endemic countries, CHWs have now important roles in malaria intervention including malaria case management, prevention including health surveillance, and health promotion specific to malaria [3]. Microscopists in Palawan prepare Giemsa-stained blood smears, identify malaria infection and species, give anti-malaria drugs if necessary, and conduct community awareness-raising activities for malaria prevention [2].

This community-based malaria control, *Kilusan Ligtas Malaria* (KLM, Tagalog: Movement Against Malaria), was launched in 1999, and since then, early diagnosis and prompt treatment have been implemented and scaled-up throughout Palawan. Consequently, the morbidity and mortality decreased year by year to reach an annual parasite index (API) in 2010 of 13.0 [1]. However, if we analyze the decrease in the API closely, it has actually remained unchanged since 2006. Now, additional strategies to further strengthen the KLM are earnestly desired in Palawan.

In this regard, we conducted three quantitative studies and suggested a new approach to accelerate the universal access of malaria patients to diagnosis, treatment, and prevention [4–6], namely the studies suggested that community awareness-raising activities of the microscopists would strengthen the effective prevention practices of the residents by increasing the likelihood of their seeking appropriate treatment. These activities could be also enhanced by additional follow-up interventions to improve service quality of the microscopists and their usage ability. However, these findings were still raising the question of how to effectively address the recent plateau in malaria incidence in the province.

Thus, in the present study, we performed a qualitative study based on the bottom-up approach to provide more profound data to strengthen this community-based malaria control from the microscopists’ points of view. The bottom-up approach could bridge the gap between proposer and practitioner by not relying on central guidance but by using the creativity and will of the people working in the community [7, 8]. Some studies have obtained findings to develop and/or evaluate strategies using qualitative data on specific CHWs’ perceptions (such as issues, barriers, or motivations), but no intervention design was discussed by applying the perceptions [9–12]. The present study explained the methods of extracting findings from qualitative data on the CHWs’ perceptions that were relevant to intervention design. We gleaned such spatiotemporal and multilateral perceptions of the CHWs as motivation for applying to be microscopists in the “Past” category; job satisfaction, role, problems, and saddest and happiest experiences working as microscopists in the “Present” category; and willingness towards task shifting in the “Future” category, and then tried to provide a path to promote a better strategy in an articulate manner.

## Methods

### Participants

The present study was a qualitative study conducted in the province of Palawan, Philippines. The population comprises various ethnicities, and the registered population was estimated to be 1,025,800 in 2010 [13]. The southern region has a much higher incidence of malaria than the northern region (Table 1).

**Table 1:** Distribution of confirmed malaria cases, API, and microscopists/region (year 2011)

Region	Confirmed malaria cases	API	Microscopists	
	(*P*. *falciparum*)		Registered	Participants
Total	4984 (76.3%)	5.71	290	50 (17.2%)
Northern region	200 (57.0%)	0.60*	115	28 (24.3%)
Puerto Princesa City	795 (71.2%)	3.84	30	0 (0%)
Southern region	3989 (78.3%)	12.1*	145	22 (15.2%)

*API* annual parasite index

*Chi-square test between the northern region and southern region (*p* < 0.0001)

Focus group discussions (FGDs) employing a total number of 50 microscopists were successfully conducted in two municipalities (Taytay and San Vicente) from the northern region in February 2011 and in two municipalities (Bataraza and Brooke’s Point) from the southern region in November 2010 (Table 2). Not only the variation in localities along the island of Palawan but also the incidence of malaria and the socio-economic status of each region were taken into consideration when choosing the municipalities to be representative study sites.

**Table 2:** Socio-demographic status of participants with respect to place of assignment

Socio-demographic status	Total (*N* = 50)	Northern region (*n* = 28)	Southern region (*n* = 22)	*p* value
Age				
Mean (SD)	38.6 (6.8)	39.2 (6.4)	37.7 (7.4)	0.451^a^
Gender				
Men	8	3	5	0.223^b^
Women	41	24	17	
Marital status				
Never married	5	2	3	0.177^b^
Married	42	26	16	
Divorced	1	0	1	
Widowed	2	0	2	
Education				
No grade completed	3	2	1	0.588^b^
Elementary	1	0	1	
High school	28	17	11	
Higher	18	9	9	
Occupation				
Homemakers	33	22	11	0.044^b^*
Farmer	12	3	9	
Other	5	3	2	
Religion				
Catholic	28	19	9	0.053^c^
Other	22	9	13	
Household wealth^1^				
Mean (SD)	2.5 (1.4)	2.3 (1.3)	2.9 (1.5)	0.107^a^
Duration of work as microscopist (months)				
Mean (SD)	94.8 (45.9)	111.9 (41.4)	73.1 (42.9)	0.002^a^**
Distance from home to health center (min)				
Mean (SD)	22.7 (30.0)	21.4 (23.2)	24.3 (37.5)	0.736^a^

*Significant place of assignment difference (0.01 < *p* < 0.05)

**Significant place of assignment difference (0.001 < *p* < 0.01)

***Significant place of assignment difference (*p* < 0.001)

^a^ANOVA

^b^Fisher’s exact test

^c^Chi-square test was used to clarify the place of assignment difference

^1^This scale scores from 1 to 8 points, with 1 point each for the following: electricity, radio, television, refrigerator, bicycle, motorcycle, bike-car, and tin or cement wall

### FGDs

The FGD method was chosen because it could stimulate group dynamics of the participants by providing the facilitator with rich data, which would not have been possible with one-to-one interviews, especially when there is little prior information on the participants [14, 15].

The FGDs were conducted in the local language (Tagalog) by facilitators who were local malaria experts with extensive experience with FGDs. During the discussions, the following open-ended questions were used to gather the broad viewpoints of the microscopists: microscopists’ perceptions of the past situation (motivation for applying), the present situation (job satisfaction, roles, problems, saddest and happiest experiences working as microscopists), and the future situation (willingness towards task shifting) (Table 3).

**Table 3:** Focus group topics and key questions

Topic			Key questions
Past	Motivation	Q1	Why did you become microscopists?
Present	Job satisfaction	Q2	Are you satisfied with your job as microscopists? Why?
	Role	Q3	How many patients do you see per week in both dry and wet seasons?
		Q4	How many of your patients were diagnosed as having malaria per week in both dry and wet seasons?
	Problems	Q5	What kind of problems did you face while performing your job as microscopists?
		Q6	Do you think there are specific ethnic groups, age groups, sexes, or any kinds of people who are likely to receive your treatment? Are there any people who are not likely to receive it?
	Experience	Q7	Please tell me about your saddest experience working as microscopists.
		Q8	Please tell me about your happiest experience working as microscopists.
Future	Task shifting	Q9	Do you want to expand your job as microscopists? If yes, in which way and how if it is not for malaria?

Audio recordings were transcribed with supervision of the FGD facilitators and analyzed by the framework analysis method using the NVivo 10 software program (QSR International Pty Ltd., Doncaster, Australia) [16]. First, each focus group discussion (FGD) session was recorded and transcribed verbatim (*transcription*). Local malaria experts translated each verbatim transcription from Tagalog into English. These transcribed discussions and field notes served as the primary text documents. All of the translated verbatim transcriptions were read thoroughly and repeatedly to become familiar with the key ideas and recurrent themes (*familiarization*). Then, the transcription was systematically coded multiple times (*coding*). Using this primal set of codes, the initial thematic framework was developed to structure, label, and define data (*identifying a thematic framework*). Based on this thematic framework, the whole transcription was re-coded (*applying the thematic framework*), and the data were charted into the appropriate parts of the framework matrix to which they related (*charting*). Finally, conclusions were drawn by using charts to define concepts, map the range and nature of the phenomena, create typologies, and find associations between themes with a view to providing explanations for the findings (*interpretation*).

## Results

**Fig. 1:**
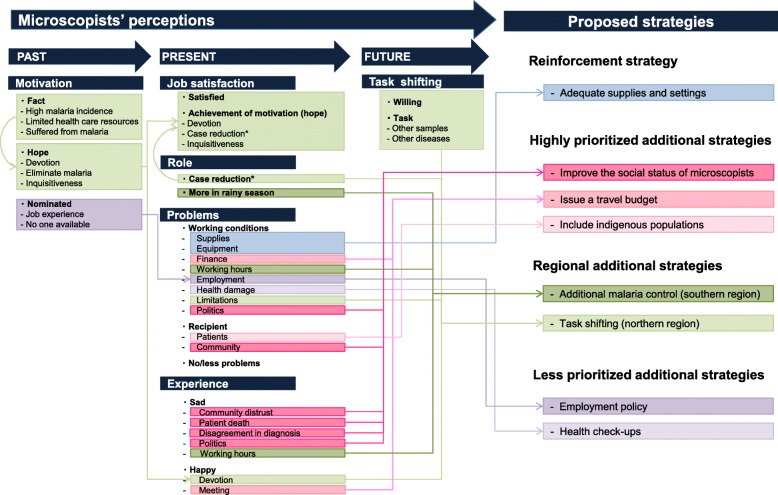
Microscopists’ perception and proposed strategies

The final results of the analytical framework of the microscopists’ perceptions of the past, present, and future are listed in Tables 4, 5, and 6, respectively. In these tables, themes, codes, and descriptions are listed by topic. Figure 1 illustrates the association of all results and the proposed strategies based on these results.

**Table 4:** Analytical framework of perception of the past (motivation)

Topic	Theme	Code	Description
Motivation	Fact	High incidence of malaria	High incidence of malaria in the villages and in Palawan.
		Limited health care resources	The health care resource is far away, and it is difficult to commute.
		Suffered from malaria	Experience of having suffered from malaria.
	Hope	Devotion	To help the community, village, and people.
		Eliminate malaria	To prevent, control, and reduce the incidence of malaria
		Inquisitive	To increase knowledge about malaria
	Nominated	Job experience	Experience to be barangay health worker
		No one available	No one was available

**Table 5:** Analytical framework of perception of the present (job satisfaction, role, problems, and experience)

Topic	Theme	Code	Description
Job satisfaction	Satisfied	Satisfied	Satisfied to be working as a microscopist.
	Achievement of the motivation (hope)	Devotion	To help the community, village, and people.
		Case reduction*	Malaria incidence is decreasing.*
		Inquisitiveness	To increase knowledge about malaria.
Role	Case reduction*	Case reduction*	Malaria incidence is decreasing.*
	More in rainy season	More in rainy season	More patients in the rainy season.
Problems	Working conditions	Supply	Shortage of materials and/or medicine.
		Setting	No electricity, broken equipment,* and/or narrowness of working space.**
		Finances	Incentives differ per municipality and are often delayed. No travel budget for home visits or official trips.
		Working hours	Patients want to be diagnosed any time, no replacement exists,* and no maternal leave was thought to be available.
		Employment	Strict recruitment policy.
		Health damage	Health problems caused by microscopy such as eye problem and headache.
		Limitations	Cannot treat other health problem.
		Politics	After election, policies often change.
	Recipients	Patient	Recurring malaria,** inappropriate intake of medicine,** and/or difficult personality.
		Community	Distrust by villagers, belief in certain religions, and/or belief in traditional medicine (indigenous residents).*
	No/fewer problems	No problems	No problems
		Fewer problems*	Along with reduction in malaria, fewer problems occur.*
Experience	Sad	Community distrust	Difficulty in being trusted by the community.
		Patient death	Patient died because the patient or their family did not trust microscopists and did not seek treatment from microscopists.
		Disagreement in diagnosis*	Diagnoses of medical technologists or private hospitals do not match with those of microscopists.*
		Politics	Autocratic behavior of politically strong persons.
		Working hours	Patient wants to be diagnosed any time, and no maternal leave was thought to be available.
	Happy	Devotion	Could help the patients and community.
		Meeting	Gather with other microscopists in meetings, training, and yearly malaria congress.

*Only mentioned in the FGDs in the northern regions

**Only mentioned in the FGDs in the southern regions

**Table 6:** Analytical framework of perception of the future (task shifting)

Topic	Theme	Code	Description
Task shifting	Willing	Willing	Willing to task shift
	Task	Other samples	Stool, urine, and sputum
		Other diseases	Parasitic disease and tuberculosis

### Motivation

Under this theme, participants in the present study indicated that their motivation was based on three themes. The first was the motivation based on facts, which were the high malaria incidence in their village, limited health care resources in their village, and to have suffered from malaria. The second theme was the motivation based on hope towards the future, the contents of which were devotion to help their village, to eliminate malaria, and inquisitiveness to learn about malaria. The third theme of motivation was that they were nominated by the village captain or midwife because of their job experience as a Barangay Health Worker (BHW) and/or because no one was available (Table 4).

Devotion was mentioned in all FGDs and was the most frequently appearing motivation. It was often mentioned along with the hope to eliminate malaria in their village where limited health service is available.

Our areas are far from the hospital, and we know that Palawan is known for malaria. So I wanted to help my fellow villagers, they (the village inhabitants) will not go to the town (where there is the hospital) just to have malaria smear to know if they have malaria or not. (Code: high malaria incidence, limited health care resources, and devotion)

Some applied because they or their family had suffered from malaria and had difficulty in getting appropriate treatment in the hospital or any health care resources, which are far from their village.

Way back in 1999, 4 of us in our family got sick, me and 3 children. During that time our *barangay* (Tagalog: village) captain was looking for someone to be trained by KLM (the administrating organization of this community-based intervention). I applied and was accepted, thinking to help our barangay. Not only us who got sick but many, that is why I applied. My child was given dextrose at RHU (rural health unit). We went home with my child with dextrose, and back again in RHU in the following day because we were not allowed to sleep at the RHU (even it is far from our village). So when I heard of the training I applied to be experienced and to give medicine. There are a lot of cases and it is difficult to commute (to the health care resource). (Code: high malaria incidence, limited health care resources, suffered from malaria, and devotion)

When no one was applying, it seems that the village captain and/or midwife nominated one of their BHW to be a microscopist. Few were forced to be microscopists but ultimately, they were pleased to be one because they were able to help their villages.

### Job satisfaction

Participants were satisfied because they could realize their motivation towards the future (hope) (Table 5). They were satisfied because they were able to help the community (devotion) and increase their knowledge about malaria (inquisitive). Some were satisfied because they could learn not only about malaria but also about other medical aspects such as immunization, taking blood pressure, measuring body weight, and assisting in feeding. This additional knowledge was related by midwifes working in the health centers where the microscopists work. Moreover, the decrease in the incidence of malaria was also an aspect that increased their satisfaction, but it was only mentioned in the northern region (less malaria).

### Role

Microscopists from the southern region where the incidence of malaria is high had many more duties and responsibilities than those from the northern region. Moreover, only the FGDs in the northern regions recognized the yearly decrease in malaria patients, whereas none in the southern regions mentioned a decrease (Table 5). The participants from both regions recognized that there were more malaria patients in the rainy season because of the increased number of insect vectors.

### Problems

Although the job satisfaction of the participants was high, it was in this section discussing their problems that they most warmed up, and it was the longest section among all of the questions. The problems mentioned in the discussions were classified into three groups: working conditions, recipients, and no/few problems (Table 5).

The most frequently appearing problem was that of working conditions, which consisted of supply, setting, finances, working hours, employment, health damage, limitations, and politics. Among these components, the delay in the purchase or supply of essential materials (mainly Giemsa stain, with a very few mentioning slides, cotton, alcohol, medicine, etc.) was most discussed.

The delay in medical supply was mentioned in both regions, but only in the northern region, in which the incidence of malaria was low and decreasing, was the problem of limited primaquine availability mentioned. If there were no cases of *Plasmodium vivax* in the previous year, then the provision of primaquine is stopped, and if there are cases of *P*. *vivax*, they have to transport the patient.

Last January there was one case of *vivax* but we did not have primaquine, because we did not have any *vivax* case the previous year. (Code: supply)

Several limitations of the setting were also mentioned. Electricity is not yet or only partly supplied in rural villages in Palawan, and limited light was causing difficulty in examining malaria especially in bad weather or at nighttime. Some participants were conducting microscopic examination in the open air under the sun. Broken microscopes and solar energy system equipment, and narrowness of the workplace were also mentioned.

In our case, we have no electricity during the day, only during the night. Yes, our place is very island (very far from the main island), sometimes those from the island will ask for examination in the night, and we need to examine right away. (Code: setting)

Among working conditions, problems related with finances were also often mentioned. The problem of incentives, which differs by municipality and which sometime causes delays, and that of the travel budget were frequently discussed, although some participants said that they were volunteers and were not interested in incentives. A travel budget was needed for official trips to make reports or attend meetings and to make home visits in households scattered about the village. Some microscopists were budgeting their own money for these trips.

With supplies, I have no problem, even in the *barangay* (Tagalog: village). My problem is the financial. I have incentive. The problem is for travel. We have monthly meeting, if we have report and the expenses in attending meeting is at our own expense. Also if there is urgent call, you have no allowance for that. If we have to refer to other hospital, we use our own money. Those patient who do not have money for the fare, it is the microscopist who will shoulder the expenses. (Code: finances)

For this travel budget problem, one microscopist had a countermeasure, which was to ask the patient to come to the health center where the microscopist works.

Yes we told them that they will be the one to come to us and then come back to give the result. So we told them that we help each other. (Code: finances)

Other problems relating to working condition were also discussed. Because patients want to be diagnosed when they want and no backup personnel exist, some participants had to examine the patient during the nighttime or holidays (working hours). Some criticized the employment conditions because of the strict recruitment policy (less than 35 years old with no foster children). Maternal leave was also thought to be not possible. Health damage, especially eye problems and headaches from using the microscope, were also mentioned. Some faced limitations because they cannot treat health problems other than malaria. Politics was also a continuing problem because after an election, new politicians often change the policies and sometime the new head tries to change the microscopist.

Problems of the recipients (patient and community) were also discussed. In the southern region where the incidence of malaria is high, recurring malaria and inappropriate intake of medicine were discussed.

In my case mam, for example the patient is positive this month then the following month positive again, then next month positive again, always coming back. (Code: patient)

There was a patient who did not take all the medicine and went back to work, and later that night he have attack of malaria and later he died. I don’t know if he had relapse or he is well according to him, but he did not finish the medication. He went back to work, but he was brought to the hospital and later he died. (Code: patient)

Community acceptance of microscopists was also a problem. Some villagers living in the mountains, mainly indigenous people, did not or continued not to trust treatment from a microscopist and were seeking treatment from an *albularyo* (Tagalog: traditional healer). Microscopists inferred that this was because they believed in traditional medicine. Some community members who believed in certain religions also refused to accept treatment from the microscopist and were treating themselves with handmade medicine.

First, the highest number of cases in San Vicente is Karuray, no. 1, the highest and many cases of malaria, and most of them are not inform because it is difficult to go to them because they believe that malaria is for the *Albularyo* (Tagalog: traditional healer) only. Mam if we come to them they will fight against us. Now we are trying to explain until some are convinced. We started at 2002 and the barangay officials are difficult to work with. (Code: community)

Several participants explained how they had changed the health-seeking behavior of these indigenous residents by teaching about malaria and the microscopist’s role. In their villages, indigenous residents come down from the mountains where they live to get a diagnosis and treatment from the microscopist.

In my area of assignment, there are some indigenous residents. They come down from the mountain. Before not and they were afraid. Now they are afraid to die in the forest. We tell them that malaria can cause death, and we can check their smear if they come down. They are still afraid of immunization. They believe that they will not be able to walk after the injection. (Code: patient)

Some participants said that they had no problems or fewer problems. The participants from the northern region said that along with the reduction in malaria, there are fewer problems occurring in relation to their work as microscopists.

In the long run mam, little by little malaria cases became lesser. And our problem is also lesser and other community members understand us, they want us to stay there. (Code: fewer problems)

### Experience

The majority of sad experiences of microscopists were related to community distrust. In the beginning of the KLM project, some microscopists faced difficulty in gaining the trust of the community (Table 5). Little by little, the community started to trust microscopists and started to get diagnosed by them. Some blamed microscopists because the patients collapsed after being pricked in the finger for blood sampling. Some patients, especially relatives of politically strong persons, blamed, and sometime tried to sue, the microscopists for misdiagnosis. However, microscopists could face these problems with confidence because they had proof of blood smears and knowledge about the diagnosis.

One day a patient came from mountain area where malaria was once epidemic but now not too much. The patient was a boy. I made a smear and was positive for *Plasmodium falciparum* 4 (high parasetemia). The father did not believe because the boy was still playing. But later the boy had convulsion. They brought him to private hospital after I diagnosed him as falciparum 4, and I have told the father to give this medicine and bring him to the hospital. Good that the boy is in good health and was also diagnosed falciparum 4 (at the private hospital). I also thought I was wrong but when he was diagnosed as falciparum 4 (at the private hospital), the father said that the microscopist knew that. Then later people started to believe my diagnosis. (Code: community distrust)

Sadly and frustratingly, some microscopists experienced the death of their malaria patients, who were not being diagnosed and treated by microscopists because the family did not trust them (patient death).

In my case, there was a patient positive with malaria who died but was brought to the hospital. I did not diagnose because they said the child is in good health but later had convulsion. They brought the child to the hospital. It was falciparum 4. Need to transfuse blood but it will come from Puerto Princesa City. When it arrived, the boy was already dead. (Code: community distrust, and patient death)

The microscopists from the northern regions sometimes had the frustrating experience of not being believed by the patient that he or she was malaria negative or positive because a medtech or private hospital provided an opposing diagnosis (disagreement in diagnosis). This disagreement was thought to occur because when microscopists first made blood smears, the parasitemia was too low to be examined; because the medicine described by microscopist had worked and the patient had no malaria when he or she received treatment from a medtech/private hospital; or because in the northern region malaria is less endemic than in the southern regions, the medtech/private hospital diagnosed the patient only from their symptoms without also checking blood smears.

Another sad experience was related to politics. Some microscopists had to resign unwillingly because the newly elected person in power wanted to change the microscopist or because there is no system for maternal leave.

Sad experiences relating to working hours were also mentioned. As basically only one microscopist is active per village and works only few days per week, some patients complained about the inconvenience. Patients want to be diagnosed and treated as soon as possible, even at nighttime or on holidays. Even though microscopists have high motivation to devote themselves to their community, it is a difficult burden to accept patients at all hours. Some microscopists found a solution by asking the BHW or midwife to take and store blood smears of the patients when the microscopist is not at the health center, and then they examined the smear later. Even with this solution, other problems occurred because of miscommunication.

Many microscopists mentioned the fact that their ability to help the patients was the happiest experience of their work as a microscopist (devotion). The following statement made by one microscopist describes her happiest experience of being able to help a person suffering from a relapse of malaria and also the fact she could better diagnose and treat malaria patients than the hospital could.

I have one patient who was always in the hospital. He was always malaria positive. One time, he approached me because he knew that I am a microscopist. Then I found out that he had *Plasmodium vivax* (which is the kind known to relapse without specific treatment). He was treated for 14 days. One month after treatment he came back and gave me chicken because it has been one month that his illness did not occur. (Code: devotion)

Microscopists also were very happy to gather with other microscopists from different municipalities during meetings, training, and the yearly malaria congress. Still, some complained because they had to spend their own money to participate in such occasions.

### Task shifting

Microscopists were willing to extend their task and to be trained to examine other samples (stool, urine, sputum) and other disease such as parasites (worms) and tuberculosis (Table 6). The motivation for this task shifting was inspired by their inquisitiveness and devotion. These were also the main motivations to become microscopists, which were related to job satisfaction.

## Discussion

In summarizing these results of the microscopists’ perceptions (Fig. 1), the present study proposed the following strategies: reinforcement strategy (adequate supplies and settings), highly prioritized additional strategies (improving social status of microscopists, issuing a travel budget, and including indigenous populations), regional additional strategies (additional malaria control in the southern region and task shifting in the northern region), and less prioritized additional strategies (employment policy and health checkups).

### Proposed strategies

#### Reinforcement strategy

First of all, as a key operation, a reinforcement strategy was suggested to avoid the occasional lack of adequate supplies and inadequate settings, which was pointed out by the microscopists. To fulfill the microscopists’ tasks, the supply of necessary materials and maintenance of the setting should be well secured. Particularly, microscopists in Palawan frequently mentioned the shortage of Giemsa stain solution because it is the only reagent being charged to the village. In fact, some microscopists had difficulty in asking the village head to purchase Giemsa stain solution. KLM should consider and find solutions to resolve this problem.

Primaquine is an essential drug for the radical cure of *P*. *vivax* patients by preventing relapses [17] and is also indispensable to the elimination of malaria in Palawan [1]*.* In 2015, about 4% of estimated cases globally were due to *P*. *vivax*, but outside the African continent, the proportion of *P*. *vivax* was 41% [18]. Despite relatively low prevalence measurements and parasetemia levels than *P*. *falciparum*, along with high proportions of asymptomatic cases, this *P*. *vivax* is not benign [19]. Without strategies regarding *P*. *vivax-*specific characteristics, progress toward world malaria elimination will not be realized. Thus, primaquine should be supplied in sufficient amounts by the KLM even in villages where *P*. *vivax* cases are not common.

In addition, in rural areas of Palawan where the electricity supply is limited, practical implementation of measures allowing the microscopists to fully perform their tasks to diagnose malaria even in bad weather or at nighttime should also be provided.

#### Highly prioritized additional strategies

Second, as a prioritized additional strategy, improvement of the microscopists’ position in society should be required. Many microscopists faced distrust by the community when they started to work. Overcoming this distrust solely through the microscopists’ quiet dedication to malaria diagnosis and treatment will not be enough. Public awareness activities to recognize the importance of the microscopic diagnosis or, indeed, the microscopists themselves are needed. Creditable microscopists in the neighborhood enabling free access of villagers to appropriate treatment will eventually acquire the trust among the local communities.

Issuing a travel budget including food allowance and travel insurance to microscopists was also proposed to help find active cases in scattered households and for other official trips. In some municipalities, microscopists were given financial incentives mainly to cover the cost of transportation, but most microscopists in other municipalities were using their own money for their travels. Most of the health centers were basically in the middle of the villages; microscopists sometimes had to cross several mountains to reach scattered households. They said that malaria in such settlements was often times a big problem because of the higher density of malaria mosquitoes. This problem of insufficient travel budget was also mentioned in our previous paper as a problem to be solved [4]. While at the same time, a study that examined different remuneration models of CHWs showed that if payment or incentives are perceived inadequately, then this can demotivate CHWs [20]. Issuing a travel budget should be carefully considered and implemented.

Moreover, many microscopists said that their happy experiences were meeting other microscopists from different municipalities in yearly malaria congresses or other meetings. Therefore, a travel budget for such purposes should be considered to maintain their job satisfaction and consequently maintain or improve the quality of their work.

The other highly prioritized additional strategy was to include indigenous people as a key target population in the KLM activities. As was found in a previous study, some indigenous people were not likely to seek diagnosis and treatment by microscopists in Palawan, and, in fact, they preferred to be treated by traditional healers, or *albularyo* in Tagalog [6]. *Albularyo* were still present in Palawan in 2015 and continued to play a salient role in the health care of some community members in the Philippines. These indigenous populations were vulnerable/at high risk for malaria infection due to the remoteness of their villages, language (Tagalog) barrier, their lack of education and persistently poor health and nutritional status, and, most importantly, their socio-cultural isolation from current government service delivery. A previous study in Palawan reported that community awareness-raising activities by microscopists for indigenous people could strengthen their ability to practice effective malaria prevention and seek appropriate treatment [5, 6]. Precisely, awareness of knowledge on malaria transmission, about which indigenous populations happen to have low knowledge, would strengthen effective prevention [5], and improving their knowledge of malaria symptoms would encourage them to seek appropriate treatment [6].

Malaria control among indigenous populations should be the key to eliminating malaria in Palawan, Philippines, as in other malaria-endemic Asian Pacific countries [11, 21]. Especially in these countries, malaria burden have been significantly reduced [18]. Moving towards malaria elimination, interventions now try to target populations at higher risk of malaria, including indigenous populations, migrants, and forest workers, who are frequently not reached by health services [22, 23].

#### Regional additional strategies

Third, additional malaria control strategies are required. In the southern region, appropriate diagnosis and treatment by the microscopists alone might not be sufficient to achieve a decrease in the incidence of malaria. Reduction of the number of malaria mosquito breeding sites [24, 25] or the use of long-lasting insecticide-treated nets or indoor residual spraying is needed [26, 27].

In the northern region, task shifting of the microscopists is proposed as a regional additional strategy because microscopists in the present study had high motivation to extend their tasks in a province in which critical shortages of highly educated health professionals are recognized. Epidemiological data also indicate a changing pattern of disease in Palawan [28], for which microscopists can play additional significant roles. With the support of midwives, it will be possible to employ highly motivated microscopists to work on several health problems other than malaria. Several studies have proved that task shifting of community health workers can contribute to addressing the insufficient health workforce worldwide [29, 30].

#### Less prioritized additional strategies

Finally, as less prioritized additional strategies, the following two items were suggested: an employment policy and the need for health checkups for them.

There might be a need to reconsider the recruitment policy. Although a “strict” policy may easily balance cost-effectiveness, a more appropriate recruitment method will make the present community-based strategy more sustainable. Although microscopic examination exposes to few health hazards, regular health checkups and/or precautions that should be taken to reduce any health problems should be prioritized at malaria congresses or yearly meetings.

### Possibility of sympathetic motivation

Along with the proposition of the four strategies as discussed above, the present results represent the general possibility that health service delivery can be strengthened and/or sustainably maintained by CHWs’ sympathetic motivation, and not by financial incentives, in resource-poor settings. The success of this community-based malaria control project was suspected to be partly because the project could satisfy the motivation of the microscopists. This led to high job satisfaction, which enhanced their work performance. Their motivation was the simple hope of devoting their work to their malarious villages. Most microscopists became microscopists because they hoped to change the reality (high incidence of malaria, limited health care resources) in their villages and gained high job satisfaction because they could contribute by diagnosing and treating malaria by themselves in their villages. The results of the present study might have been due to the microscopists’ characteristics, such as subsistence status (mainly homemakers), religion, and a stable household financial status, and therefore might not be applicable to all resource-poor settings. However, for example, a previous study targeting the same microscopists supports the impact of job satisfaction on work performance [4]. Job satisfaction of the microscopists and other CHWs should be the subject of greater attention and emphasis in order to strengthen sustainable health service delivery.

### Limitation

Although the FGD method can provide rich data especially when there is little prior information on the participants, there are some methodological limitations. Unsuitable topics and views exist for the FGD methods. Therefore, it is difficult to suggest a strong recommendation. However, it is also true that the discussions in the FGDs highlighted some dimensions among CHWs’ perceptions. Therefore, the proposed strategies are expected to be certainly practical and effective to strengthen the community-based malaria control.

Moreover, the participants of each FGD were around 12. This number was almost the upper limit of the number of participants that were conventionally involved in productive FGDs. To decrease the risk of respondents’ being hidden in the crowd and dropping their inputs, the facilitators conducted the FGDs very carefully to get sufficient replies from all the participants.

## Conclusion

This qualitative study was based on the bottom-up approach using CHWs’ (microscopists) perceptions about their past, present, and future to propose practical and effective additional strategies. It was proven important to understand profound data such as realities or barriers that would help to strengthen the community-based malaria control.
